# Preoperative Multiparametric MRI‐Based Tumour–Periprostatic Adipose Tissue Interface Characterisation for Extraprostatic Extension Prediction in Prostate Cancer

**DOI:** 10.1002/cam4.71613

**Published:** 2026-02-05

**Authors:** Subo Zhang, Leiming Huo, Zhitao Zhu, Jinxin Wan, Lei Xu, Jiabao Xia, Yongjun Xu, Jingfang Liu, Yan Zhao

**Affiliations:** ^1^ Department of Medical Imaging The Second People's Hospital of Lianyungang Lianyungang Jiangsu China; ^2^ Department of Medical Imaging Lianyungang Clinical College of Jiangsu University Lianyungang Jiangsu China; ^3^ Department of Medical Imaging The Second People's Hospital of Lianyungang Affiliated With Kangda College of Nanjing Medical University Lianyungang Jiangsu China; ^4^ Department of Respiratory The Second People's Hospital of Lianyungang Lianyungang Jiangsu China

**Keywords:** extraprostatic extension, interface features, multiparametric magnetic resonance imaging, periprostatic adipose tissue, prediction model, prostate cancer

## Abstract

**Objective:**

To evaluate the independent predictive value of tumour–periprostatic adipose tissue (PPAT) interface features on preoperative multiparametric magnetic resonance imaging (mpMRI) for extraprostatic extension (EPE) in prostate cancer and to compare discrimination and clinical net benefit with a baseline clinical model.

**Methods:**

This single‐centre retrospective cohort included patients who underwent radical prostatectomy with mpMRI completed within 8 weeks. On a single axial slice at maximum tumour diameter, five simplified interface features were measured using standard PACS tools: contact length, contact angle, T2 signal intensity ratio, interface apparent diffusion coefficient (3‐mm annular zone) and capsular integrity score (0–2 scale). A baseline clinical model (prostate‐specific antigen [PSA], PSA density, PI‐RADS and biopsy Gleason score) and a combined model (baseline variables plus LASSO‐selected interface features) were constructed. Bootstrap internal validation (1000 iterations) with bias correction was performed. Discrimination was assessed using the area under the curve (AUC), and calibration curves and decision curve analysis evaluated accuracy and net clinical benefit.

**Results:**

A total of 240 patients were included, with an EPE prevalence of 34.2% (82/240). The combined model achieved a bias‐corrected AUC of 0.823 (95% confidence interval [CI]: 0.768–0.878), suggesting improvement over the baseline model's AUC of 0.744 (95% CI: 0.680–0.808). Decision curve analysis revealed a higher net benefit for the combined model across clinically relevant threshold probabilities (10%–50%).

**Conclusions:**

Simplified tumour–PPAT interface features independently predict EPE without increasing imaging complexity, improving discrimination and clinical value for preoperative risk stratification.

## 1 Introduction

Extraprostatic extension (EPE) represents a critical prognostic indicator in prostate cancer, considerably influencing surgical planning, treatment strategy, and biochemical recurrence risk. The accurate preoperative prediction of EPE remains essential for determining optimal surgical margins, selecting candidates for nerve‐sparing procedures, and identifying patients who may benefit from adjuvant therapy [1, 2]. Current clinical nomograms incorporating prostate‐specific antigen (PSA), the Gleason score, and the clinical stage provide reasonable predictive accuracy yet demonstrate suboptimal sensitivity for EPE detection, particularly in patients at intermediate risk [3, 4].

Multiparametric magnetic resonance imaging (mpMRI) has emerged as the cornerstone of prostate cancer staging, with established imaging features such as capsular bulge, irregular capsular contour and apparent capsular breach serving as qualitative indicators of EPE [5, 6]. However, the subjective nature of these assessments contributes to significant inter‐observer variability and limited reproducibility [7]. Quantitative imaging biomarkers, particularly tumour capsular contact length measured on T2‐weighted images (T2WIs), have demonstrated improved consistency, with meta‐analyses reporting pooled sensitivities of 57%–71% and specificities of 81%–92% for EPE detection [8, 9]. Nevertheless, contact length alone fails to capture the complex spatial and biological interactions occurring at the tumour–capsular interface [10, 11]. Experimental evidence demonstrates that adipocyte‐secreted CCL7 promotes tumour cell migration through CCR3‐mediated signalling, with this effect amplified in obesity, facilitating extraprostatic dissemination [12]. Furthermore, adipose stem cells within periprostatic adipose tissue (PPAT) contribute to the tumour microenvironment, modulating cancer cell behaviour through paracrine mechanisms [13]. These biological insights suggest that the interface between tumour and PPAT represents a critical zone of interaction that may harbour predictive imaging signatures beyond simple contact measurements.

Radiomics‐based approaches have shown promise in EPE prediction by extracting high‐dimensional texture and intensity features from medical images [14, 15]. However, the complexity of radiomics workflows, computational demands, and challenges in reproducibility across different scanners and acquisition protocols have limited their translation to routine clinical practice [16]. Recent systematic reviews indicate that although radiomics models achieve moderate discriminative performance (pooled area under the curve [AUC] is approximately 0.80), methodological heterogeneity and lack of external validation are major concerns [17, 18].

Building upon these observations, we hypothesise that focused characterisation of the tumour–PPAT interface using simplified, clinically feasible imaging features may provide independent predictive value for EPE. Previous studies examining PPAT have primarily assessed gross morphometric parameters such as PPAT thickness or area, yielding inconsistent results with weak correlations to PSA and paradoxical protective trends in multivariable analyses [19, 20]. These findings suggest that static PPAT measurements fail to capture the dynamic tumour–fat interaction. In contrast, interface‐specific features—quantifying the extent, geometry, and tissue characteristics of the direct tumour–PPAT contact zone—may better reflect the biological processes underlying capsular breach and extraprostatic invasion.

The objective of this study is to evaluate whether simplified tumour–PPAT interface features, measurable on a single axial MRI slice using standard PACS tools, can independently predict EPE and enhance the discriminative performance and clinical utility of baseline clinical models. By focusing on a limited set of interpretable interface parameters derived from T2WI and diffusion‐weighted imaging (DWI)/apparent diffusion coefficient (ADC) sequences, we aim to develop a practical tool suitable for preoperative risk stratification and surgical planning in patients with clinically localised prostate cancer.

## 2 Methods

### 2.1 Study Design and Ethics

This single‐centre retrospective cohort analysis consecutively enrolled patients who underwent radical prostatectomy (RP) between January 2019 and December 2024 at our institution, with preoperative mpMRI completed within 8 weeks before surgery. A total sample size of 240–300 patients was planned to ensure adequate statistical power for multivariable modelling, guided by the events per variable (EPV) criterion recommending a minimum of 10 outcome events per predictor variable [21]. The study adhered to the principles of the Declaration of Helsinki and received approval from the institutional ethics committee. Given the retrospective nature, written informed consent was waived.

### 2.2 Study Population

Patients were considered eligible if they met the following criteria: (1) histopathologically confirmed prostatic adenocarcinoma on systematic or targeted biopsy; (2) underwent RP with complete pathological evaluation; (3) had preoperative mpMRI performed ≤ 8 weeks before surgery; (4) mpMRI examination included evaluable T2WI and DWI/ADC sequences with adequate image quality; and (5) complete clinical data were available including serum PSA level, prostate volume, PI‐RADS v2.1 assessment score and biopsy‐determined Gleason score. The exclusion criteria included the following: (1) prior local treatment for prostate cancer, (2) MRI image quality deemed inadequate, (3) multifocal disease where the index lesion could not be clearly identified, (4) missing critical clinical data and (5) non‐adenocarcinoma histology.

The preferred surgical approach at our institution was robot‐assisted laparoscopic RP, performed in 87% (209/240) of cases. The remaining cases underwent conventional laparoscopic (8%, 19/240) or open retropubic (5%, 12/240) RP based on surgeon preference and patient characteristics.

### 2.3 Extraprostatic Extension Definition and Reference Standard

EPE was defined pathologically as (1) tumour extension beyond the prostatic pseudocapsule into periprostatic soft tissue or (2) tumour involvement of the neurovascular bundle structures in the absence of an intervening layer of benign prostatic tissue [22]. All pathological specimens were reviewed by two experienced genitourinary pathologists blinded to clinical and imaging findings.

### 2.4 Magnetic Resonance Imaging Acquisition and Image Processing

All mpMRI examinations were performed using 3.0 Tesla MRI systems (Siemens Healthineers or GE Healthcare) following PI‐RADS v2.1 technical recommendations. The standard protocol included high‐resolution T2WI in three orthogonal planes, DWI with multiple b‐values (typically *b* = 0, 800–1000 and 1400–2000 s/mm^2^) and calculated ADC maps. Image quality assessment was performed independently by two fellowship‐trained radiologists with 5 and 8 years of experience in prostate MRI interpretation, respectively, using a three‐point qualitative scale. Only examinations receiving acceptable or excellent quality ratings from both readers were included.

### 2.5 Tumour Identification and Interface Measurement

**FIGURE 1:**
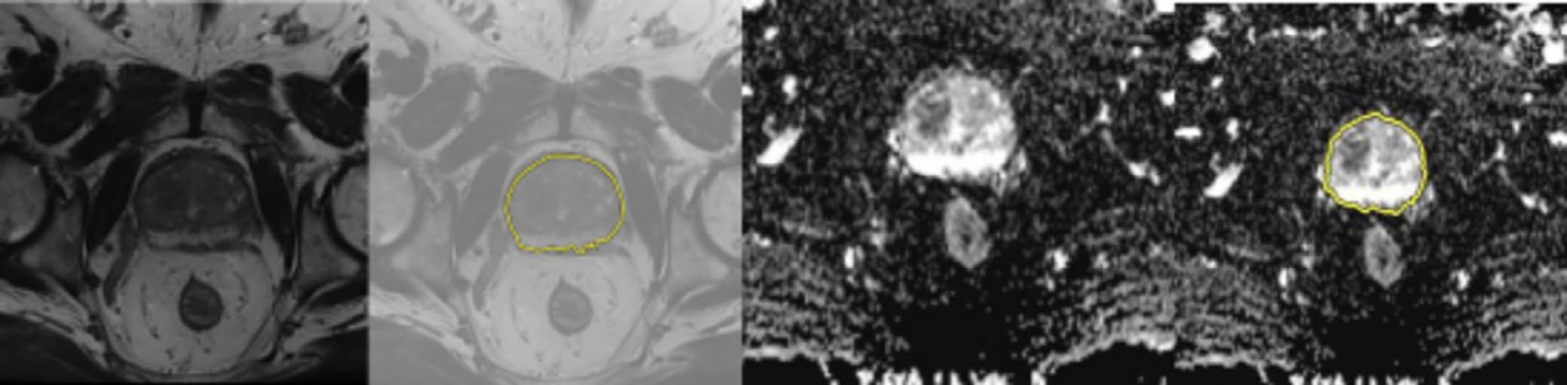
Tumour ROI delineation at maximum lesion diameter. Semi‐automated tumour segmentation workflow demonstrated on axial imaging at the level of maximal lesion diameter. Left panels: T2‐weighted imaging showing original image and image with tumour ROI delineation. Right panels: Corresponding ADC map showing original image and registered tumour ROI.

All interface measurements were performed on a single axial slice corresponding to the maximum diameter of the index lesion on T2WI. Reader A (8 years of prostate MRI experience) identified the index lesion according to PI‐RADS v2.1 criteria and selected the axial slice demonstrating the largest cross‐sectional tumour area. To assess inter‐observer reproducibility, Reader B (5 years of experience) independently performed measurements on a randomly selected subset of 30 cases (12.5% of the cohort). The tumour region of interest (ROI) delineation on T2WI and corresponding identification on co‐registered ADC maps is illustrated in Figure 1. The straightforward measurement approach on a single slice typically required 3–5 min per case.

### 2.6 Quantitative Interface Features

Five interpretable interface features were extracted, all measured on the single axial slice showing maximum tumour diameter. Geometric features included (1) contact length, defined as the linear extent (in millimetres) of the arc segment where the tumour directly abuts PPAT along the capsular boundary (measured using electronic calliper tools on the PACS workstation) and (2) contact angle, calculated as the angle in degrees subtended at the tumour geometric centroid by the arc of tumour capsular contact, with reference lines drawn from the centroid to the endpoints of the contact segment.

Intensity‐based features captured signal characteristics at the interface zone. The T2 signal intensity ratio was computed as the mean T2WI signal intensity in a small ROI placed immediately adjacent to the tumour along the contact interface (tumour side of the capsule) divided by the mean T2WI signal intensity in a symmetric region of preserved periprostatic fat on the contralateral side at the same axial level. For ADC measurements, a 3‐mm‐wide annular ROI was manually drawn to encompass the interface zone along the segment of tumour capsular contact, and the mean ADC value within this zone was recorded in units of ×10^−3^ mm^2^/s.

**FIGURE 2:**
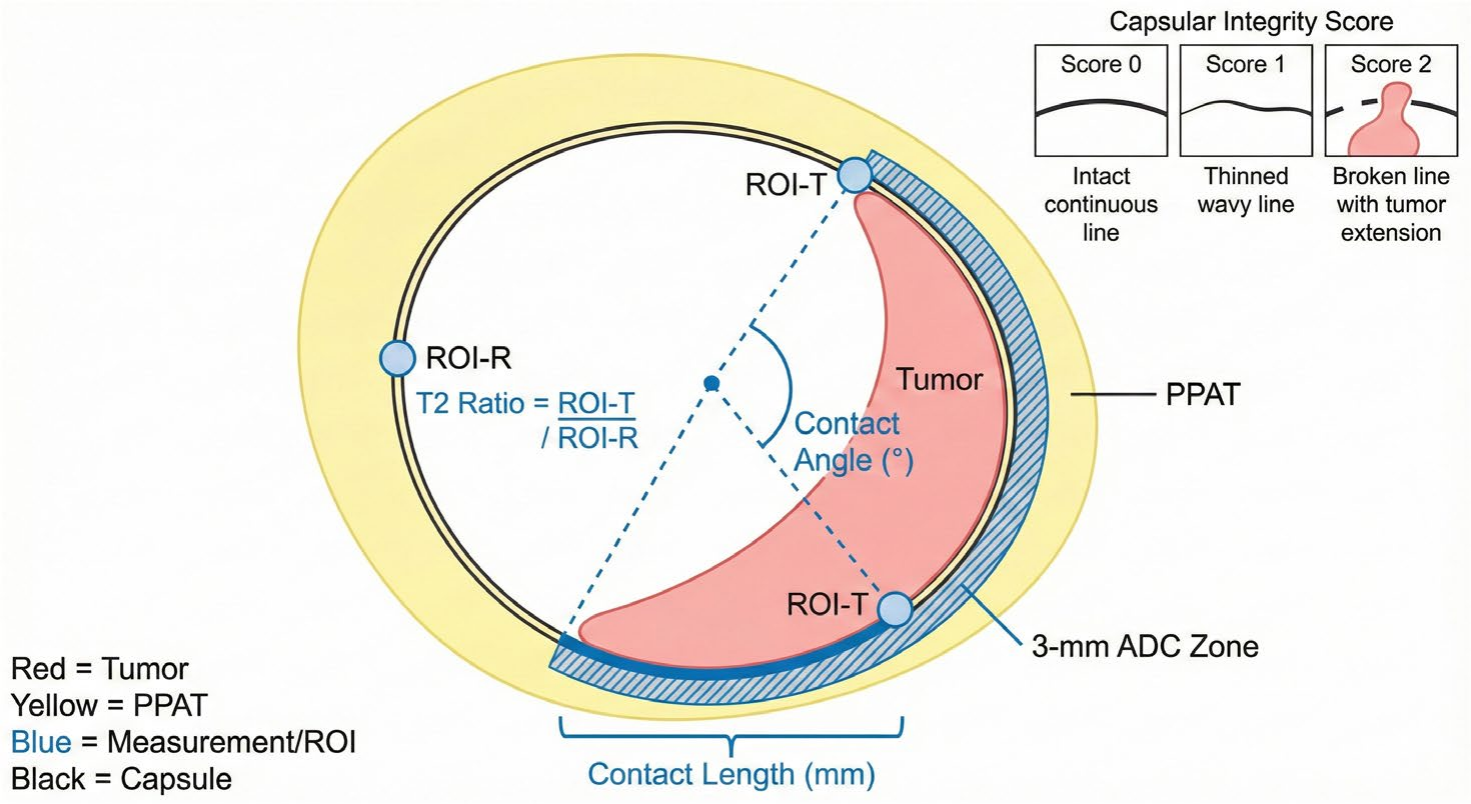
Detailed illustration of interface feature measurement methodology. Schematic diagram demonstrating the measurement techniques for all five interface features on a single axial slice at maximum tumour diameter.

Finally, capsular integrity was subjectively assessed on a three‐point ordinal scale as follows: 0 = capsule appears intact, 1 = capsule appears thinned or possibly interrupted, and 2 = capsule is clearly interrupted with the tumour extending into periprostatic fat. A detailed illustration of all ROI placements and measurement techniques is provided in Figure 2, demonstrating the standardised approach for contact length measurement, contact angle calculation, T2 signal intensity ratio ROI placement, and the 3‐mm annular ADC measurement zone.

### 2.7 Baseline Clinical Variables

The baseline clinical model incorporated four well‐established predictors: (1) serum PSA level (ng/mL), (2) PSA density (PSAD) (ng/mL/cm^3^), (3) PI‐RADS assessment category of the index lesion, and (4) biopsy Gleason score or ISUP grade group.

### 2.8 Statistical Analysis

All analyses were performed using R software (version 4.2.0). Model development proceeded in two stages: first, a baseline clinical model was constructed using multivariable logistic regression; second, the five candidate interface features underwent LASSO feature selection using 10‐fold cross‐validation with the 1‐SE rule, and selected features were combined with baseline variables in a final multivariable logistic regression model. Bootstrap resampling (1000 iterations) was employed for internal validation. Discrimination was assessed using the AUC with 95% confidence intervals (CIs), and models were compared using the DeLong test. Calibration was evaluated using the calibration slope, intercept, Hosmer–Lemeshow test, and Brier score. Clinical utility was assessed using decision curve analysis (DCA).

## 3 Results

### 3.1 Study Population and Baseline Characteristics

**FIGURE 3:**
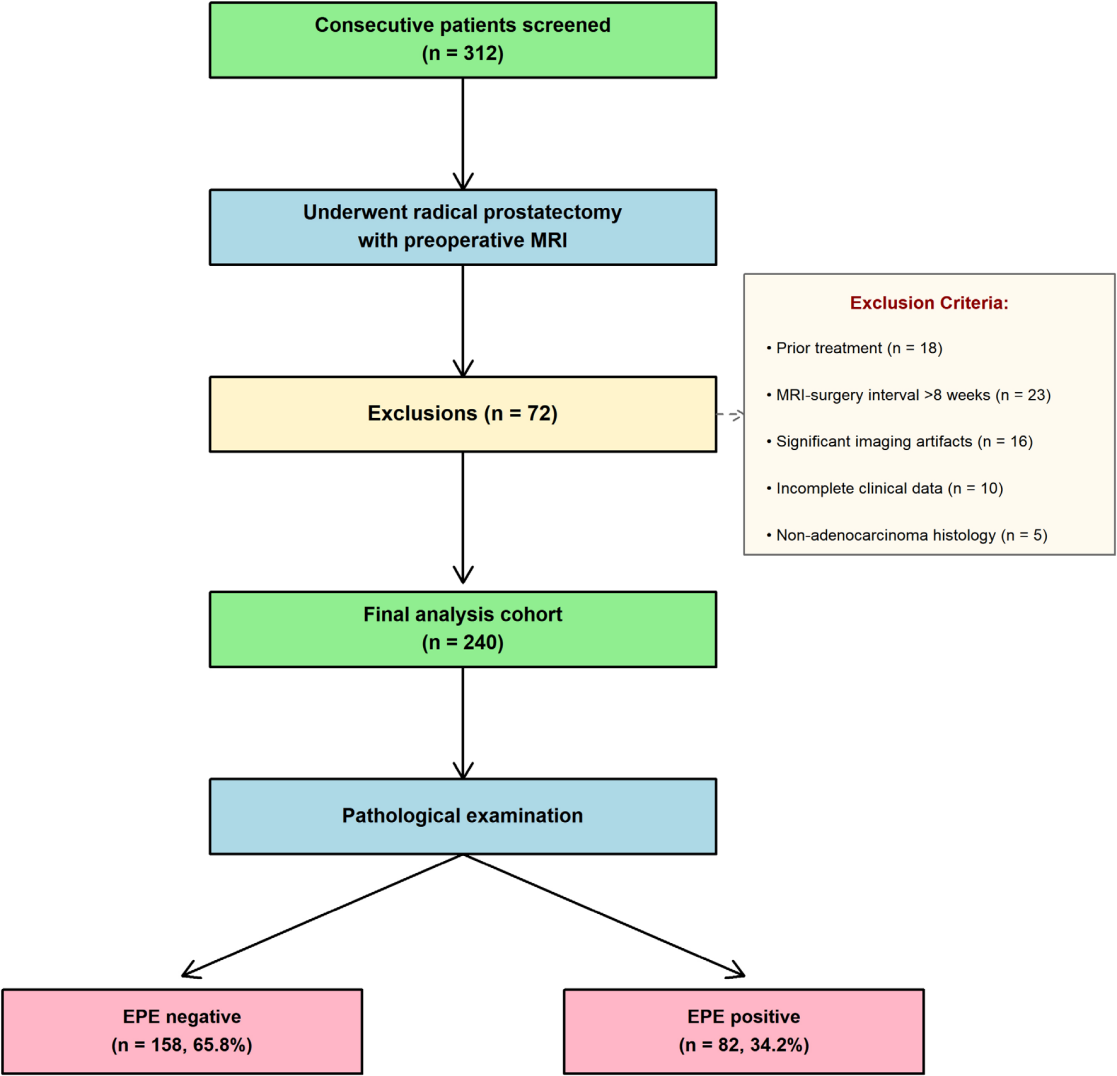
Patient selection flowchart. Diagram showing screening, inclusion, and exclusion criteria flow. Initial screening identified 312 consecutive patients who underwent radical prostatectomy during the study period. Exclusions: Prior treatment (*n* = 18), MRI‐to‐surgery interval > 8 weeks (*n* = 23), significant imaging artefacts (*n* = 16), incomplete clinical data (*n* = 10), non‐adenocarcinoma histology (*n* = 5). Final analysis cohort: 240 patients, with 82 (34.2%) demonstrating pathological EPE.

The patient selection flowchart is depicted in Figure 3. Initial screening identified 312 consecutive patients who underwent RP between January 2019 and December 2024. After applying the exclusion criteria, 72 patients were excluded for the following reasons: 18 patients had received prior local treatment, 23 patients had MRI‐to‐surgery intervals exceeding 8 weeks, 16 patients had major imaging artefacts, 10 patients had incomplete clinical data, and 5 patients had non‐adenocarcinoma histology. The final analysis cohort comprised 240 patients.

Among the 240 included patients, pathological examination revealed EPE in 82 cases, yielding a prevalence of 34.2%. Baseline clinical and imaging characteristics stratified by EPE status are presented in Table 1. Patients with EPE tended to be slightly older (mean age: 66.2 ± 7.3 years vs. 63.8 ± 7.9 years, *p* = 0.026), had significantly higher median PSA levels (15.6 ng/mL [interquartile range (IQR): 9.8–24.3] vs. 9.4 ng/mL [IQR: 6.2–14.7], *p* < 0.001) and higher PSAD (0.38 ng/mL/cm^3^ [IQR: 0.24–0.58] vs. 0.22 ng/mL/cm^3^ [IQR: 0.15–0.34], *p* < 0.001) than those without EPE.

**TABLE 1:** Baseline characteristics and comparison by EPE status.

Variable	Overall (*n* = 240)	EPE negative (*n* = 158)	EPE positive (*n* = 82)	Statistic	*p*
Age (years), mean ± SD	64.6 ± 7.7	63.8 ± 7.9	66.2 ± 7.3	*t* = 2.27	0.026
PSA (ng/mL), median (IQR)	11.4 (7.2–18.3)	9.4 (6.2–14.7)	15.6 (9.8–24.3)	*Z* = −4.82	< 0.001
Prostate volume (mL), median (IQR)	42.6 (32.8–56.4)	43.2 (33.6–58.1)	41.3 (31.4–53.7)	*Z* = −1.34	0.18
PSAD (ng/mL/cm^3^), median (IQR)	0.27 (0.17–0.43)	0.22 (0.15–0.34)	0.38 (0.24–0.58)	*Z* = −5.16	< 0.001
PI‐RADS 3/4/5, *n* (%)	23/103/114 (9.6/42.9/47.5)	21/79/58 (13.3/50.0/36.7)	2/24/56 (2.4/29.3/68.3)	*χ* ^2^ = 28.4	< 0.001
Biopsy Gleason ≥ 8, *n* (%)	70 (29.2)	30 (19.0)	40 (48.8)	*χ* ^2^ = 24.1	< 0.001
Contact length (mm), median (IQR)	13.8 (8.6–19.7)	11.2 (7.3–16.4)	18.4 (12.6–24.8)	*Z* = −5.94	< 0.001
Contact angle (°), median (IQR)	64.2 (41.5–96.8)	52.3 (34.2–78.6)	87.5 (58.3–126.7)	*Z* = −5.37	< 0.001
Interface ADC (×10^−3^ mm^2^/s), mean ± SD	0.90 ± 0.18	0.94 ± 0.16	0.82 ± 0.18	*t* = 5.18	< 0.001
GLCM contrast, mean ± SD	48.3 ± 14.7	44.6 ± 13.2	55.1 ± 15.3	*t* = −5.34	< 0.001
GLCM entropy, mean ± SD	2.73 ± 0.42	2.64 ± 0.38	2.89 ± 0.45	*t* = −4.51	< 0.001

*Note:* Capsular integrity score: 0 = intact capsule, 1 = equivocal thinning/interruption, 2 = clear capsular disruption.

Abbreviations: ADC, apparent diffusion coefficient; EPE, extraprostatic extension; IQR, interquartile range; PI‐RADS, Prostate Imaging‐Reporting and Data System; PSA, prostate‐specific antigen; PSAD, PSA density; SD, standard deviation.

The distribution of PI‐RADS scores differed markedly between groups, with 73.2% of patients with EPE having PI‐RADS 5 lesions compared with 31.6% of patients without EPE (*p* < 0.001). Similarly, high‐grade disease on biopsy (Gleason ≥ 8 or ISUP grade group ≥ 4) was significantly more common in the EPE group than in the non‐EPE group (48.8% vs. 19.0%, *p* < 0.001). Regarding interface features, patients with EPE demonstrated significantly longer tumour capsular contact length (median 18.4 mm [IQR: 12.6–24.8] vs. 11.2 mm [IQR: 7.3–16.4], *p* < 0.001) and a wider contact angle (median 87.5° [IQR: 58.3–126.7] vs. 52.3° [IQR: 34.2–78.6], *p* < 0.001) than those without EPE. Interface ADC values were significantly lower in the EPE group than in the non‐EPE group (mean 0.82 ± 0.18 × 10^−3^ mm^2^/s vs. 0.94 ± 0.16 × 10^−3^ mm^2^/s, *p* < 0.001). The T2 signal ratio also differed between groups (1.12 ± 0.21 vs. 0.98 ± 0.18, *p* < 0.001). Notably, capsular integrity scores were significantly higher in patients with EPE, with 68.3% (56/82) showing a score 2 compared with 21.5% (34/158) in patients without EPE (*p* < 0.001).

### 3.2 Feature Reproducibility and Correlations

Inter‐observer reproducibility analysis based on the 30 duplicate measurements is summarised in Table 2. Geometric features demonstrated excellent agreement, with intraclass correlation coefficient (ICC) values of 0.92 (95% CI: 0.85–0.96) for contact length and 0.89 (95% CI: 0.80–0.94) for contact angle. Interface ADC mean showed good to excellent reproducibility (ICC = 0.87, 95% CI: 0.76–0.93), whereas the T2 signal ratio exhibited good agreement (ICC = 0.81, 95% CI: 0.67–0.90). The subjective capsular integrity score demonstrated substantial to almost perfect agreement between readers (weighted Cohen's *κ* = 0.78, 95% CI: 0.63–0.93).

**TABLE 2:** Inter‐observer agreement for interface features (*n* = 30).

Feature	ICC/*κ* (95% CI)	Agreement level
Contact length	0.92 (0.85–0.96)	Excellent
Contact angle	0.89 (0.80–0.94)	Good‐Excellent
T2 signal ratio	0.81 (0.67–0.90)	Good
Interface ADC mean	0.87 (0.76–0.93)	Good‐Excellent
Capsular integrity score (ordinal)	*κ*w = 0.78 (0.63–0.93)	Substantial‐Almost Perfect

Abbreviations: ADC, apparent diffusion coefficient; CI, confidence interval; ICC, intraclass correlation coefficient; *κ*w, weighted Cohen's kappa.

**FIGURE 4:**
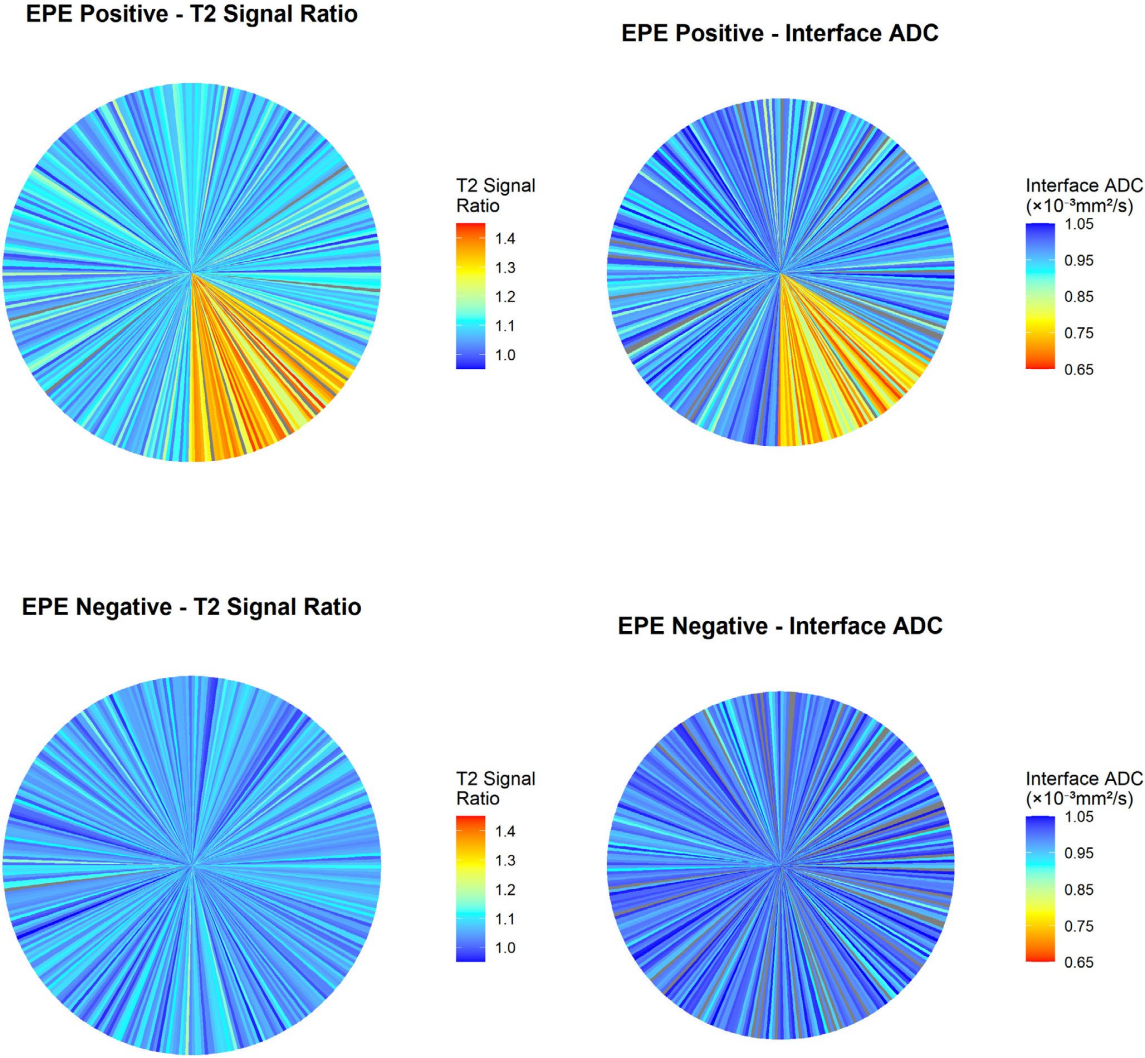
Interface parameter spatial distribution heatmap. Polar coordinate representation showing circumferential distribution of T2 signal intensity ratio and interface ADC values around the prostatic capsule in representative EPE‐positive and EPE‐negative cases. Colour scale from blue (low values) to red/orange (high values). EPE‐positive case (top row) shows focal sectors with elevated T2 ratio and markedly reduced ADC (warm colours) corresponding to sites of pathological capsular breach. EPE‐negative case (bottom row) demonstrates relatively uniform, higher ADC distribution (cool colours) consistent with preserved periprostatic fat without tumour infiltration. Each radial segment represents interface measurements at different angular positions around the tumour perimeter.

Figure 4 displays polar coordinate heatmaps illustrating the circumferential distribution of T2 signal intensity ratio and interface ADC values around the prostatic capsule in EPE‐positive and EPE‐negative representative cases.

### 3.3 Model Development and Internal Validation

Four interface features with nonzero coefficients were identified via LASSO regression: contact length, contact angle, interface ADC mean, and capsular integrity score. The T2 signal ratio was shrunk to zero and excluded from the final combined model. Coefficient estimates and odds ratios from the fitted model are presented in Table 3.

**TABLE 3:** Multivariable logistic regression coefficients (combined model).

Variable	Odds ratio (95% CI)	*p*
PSA (log‐transformed)	1.48 (0.96–2.29)	0.078
PSAD (per 0.1 unit)	1.67 (1.28–2.18)	< 0.001
PI‐RADS (per category)	2.85 (1.74–4.67)	< 0.001
Gleason ≥ 8 (vs. ≤ 7)	1.83 (0.97–3.45)	0.062
Contact length (per 10 mm)	2.14 (1.48–3.11)	< 0.001
Contact angle (per 30°)	1.67 (1.28–2.18)	< 0.001
Interface ADC (per 0.1 decrease)	1.83 (1.32–2.54)	< 0.001
Capsular integrity score (per point)	3.24 (2.15–4.88)	< 0.001

*Note:* Features selected by LASSO with nonzero coefficients.

Abbreviations: ADC, apparent diffusion coefficient; CI, confidence interval; PI‐RADS, Prostate Imaging‐Reporting and Data System; PSA, prostate‐specific antigen; PSAD, PSA density.

In the combined model, all four selected interface features demonstrated independent associations with EPE after adjustment for clinical variables. For every 10‐mm increase in contact length, the odds of EPE increased by 2.14‐fold (95% CI: 1.48–3.11, *p* < 0.001). Each 30° increase in contact angle was associated with an odds ratio of 1.67 (95% CI: 1.28–2.18, *p* < 0.001). A lower interface ADC mean significantly predicted EPE, with each 0.1 × 10^−3^ mm^2^/s decrease corresponding to an odds ratio of 1.83 (95% CI: 1.32–2.54, *p* < 0.001). Capsular integrity score was strongly associated with EPE, with each one‐point increase conferring an odds ratio of 3.24 (95% CI: 2.15–4.88, *p* < 0.001).

Bootstrap internal validation with 1000 iterations yielded optimism‐corrected performance estimates (Table 4). The combined model achieved a bias‐corrected AUC of 0.823 (95% CI: 0.768–0.878), representing a statistically significant improvement over the baseline clinical model's AUC of 0.744 (95% CI: 0.680–0.808; DeLong test *p* = 0.003). The combined model had a calibration slope of 0.96 and a calibration intercept of −0.05, with the Hosmer–Lemeshow test yielding a nonsignificant *p*‐value of 0.38. The Brier score for the combined model was 0.176, lower than the baseline model's 0.204.

**TABLE 4:** Model performance, calibration, and net benefit (bootstrap‐corrected).

Metric	Baseline model	Combined model
AUC (95% CI)	0.744 (0.680–0.808)	0.823 (0.768–0.878)^a^
Calibration slope	0.92	0.96
Calibration intercept	−0.08	−0.05
Hosmer‐Lemeshow test *p*	0.46	0.38
Brier score	0.204	0.176
Net benefit at threshold probability		
10%	0.214	0.254
15%	0.197	0.241
20%	0.178	0.224
25%	0.156	0.203
30%	0.131	0.168
35%	0.104	0.142
40%	0.076	0.109
45%	0.051	0.082
50%	0.028	0.057

*Note:* Net benefit values represent benefit per patient relative to ‘treat none’ strategy.

Abbreviations: AUC, area under the receiver operating characteristic curve; CI, confidence interval.

^a^ DeLong test comparing combined vs. baseline model: *p* = 0.003.

Using the Youden‐index‐determined optimal threshold of 0.32, the combined model achieved a sensitivity of 78.0% (95% CI: 67.5%–86.1%), specificity of 76.6% (95% CI: 69.2%–82.9%), positive predictive value of 62.1% (95% CI: 52.1%–71.4%), negative predictive value of 87.7% (95% CI: 81.2%–92.5%) and an overall accuracy of 77.1% (95% CI: 71.2%–82.3%). The confusion matrix and detailed performance metrics are presented in Table 5.

**TABLE 5:** Diagnostic performance at optimal threshold (Youden index).

	Pathological EPE+	Pathological EPE−
Predicted EPE+	TP = 64	FP = 37
Predicted EPE−	FN = 18	TN = 121

*Note:* Optimal threshold: 0.32 predicted probability. Sensitivity: 78.0% (95% CI: 67.5%–86.1%). Specificity: 76.6% (95% CI: 69.2%–82.9%). Positive Predictive Value: 62.1% (95% CI: 52.1%–71.4%). Negative Predictive Value: 87.7% (95% CI: 81.2%–92.5%). Accuracy: 77.1% (95% CI: 71.2%–82.3%).

Abbreviations: EPE, extraprostatic extension; FN, false negative; FP, false positive; TN, true negative; TP, true positive.

### 3.4 Clinical Net Benefit Analysis

**FIGURE 5:**
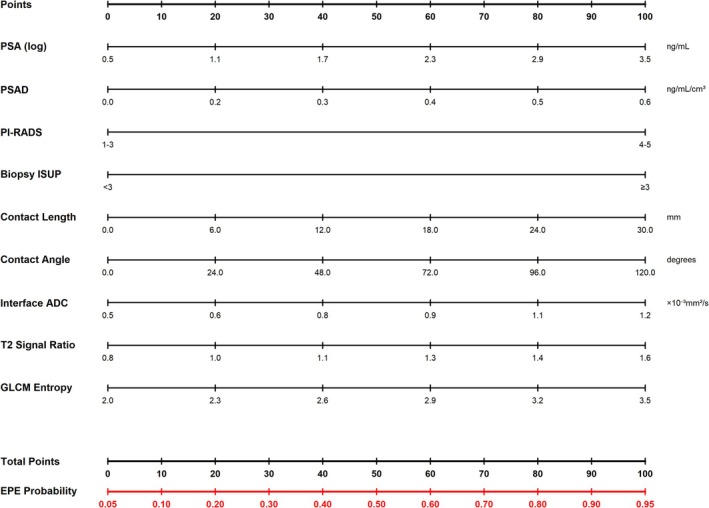
Clinical nomogram for EPE prediction (combined model). Point‐based nomogram for bedside estimation of EPE probability using the combined clinical‐interface model. Each predictor variable (PSA, PSAD, PI‐RADS score, Biopsy ISUP grade group, contact length, contact angle, interface ADC, and capsular integrity score) has its own scale in the nomogram.

The DCA results, tabulated at 5% increments of threshold probability from 10% to 50%, are shown in Table 4. At all assessed threshold probabilities within the clinically relevant range, the combined model demonstrated higher net benefit than the baseline model and the default ‘treat all’ or ‘treat none’ strategies. Based on these findings, a simplified clinical nomogram is presented in Figure 5.

### 3.5 Subgroup and Sensitivity Analyses

Subgroup analyses by PI‐RADS category revealed that the added value of interface features was most pronounced in PI‐RADS 4 lesions (combined model AUC = 0.801 vs. baseline AUC = 0.695, *p* = 0.008), with benefit remaining present but less marked in PI‐RADS five cases (combined AUC = 0.785 vs. baseline AUC = 0.721, *p* = 0.042). Stratification by Gleason grade showed consistent improvement with the combined model across all grade categories. Sensitivity analysis excluding cases acquired on different scanner vendors showed no significant performance differences. Finally, refitting the models after excluding the subjective capsular integrity score yielded minimal change in performance (combined model AUC = 0.798 vs. 0.823 with the capsular score included).

## 4 Discussion

This study suggests that simplified quantitative features characterising the tumour–PPAT interface on preoperative mpMRI, measured on a single axial slice, independently predict EPE in patients with prostate cancer undergoing RP. The combined clinical–interface model achieved significantly improved discrimination (AUC = 0.823) compared with a baseline model incorporating standard clinical variables (AUC = 0.744), and this improvement translated to meaningful clinical benefit as evidenced by DCA. Our findings support the concept that focused interrogation of the tumour–PPAT boundary using straightforward two‐dimensional (2D) measurements provides incremental prognostic information beyond conventional staging parameters, potentially informing more personalised surgical planning and treatment selection.

The superior performance of the combined model likely reflects the biological significance of the tumour–PPAT interface as a site of dynamic interaction between cancer cells and the adjacent adipose microenvironment. Mounting evidence indicates that PPAT is not merely passive adipose tissue but an active participant in prostate cancer progression through paracrine signalling, inflammatory modulation and provision of energy substrates. Laurent et al. [12] demonstrated that adipocytes within PPAT secrete the chemokine CCL7, which diffuses through the prostatic capsule and promotes migration of CCR3‐expressing tumour cells towards the adipose compartment, with this effect amplified in obese individuals. Our observation of lower ADC values at the tumour–PPAT interface in EPE‐positive cases may capture this biological process, as increased cellularity from migrating tumour cells or reactive inflammatory infiltrates would restrict diffusion. This finding aligns with broader evidence demonstrating the diagnostic and prognostic importance of peritumoural zones across various malignancies. For example, Maiuro et al. [23] recently showed that kurtosis (K)‐based MRI analysis of the peritumoural region in endometrial cancer provides valuable diagnostic information, highlighting the general principle that tumour–tissue interfaces represent biologically active zones worthy of focused imaging interrogation. Similarly, the strong association of capsular integrity score with EPE validates the radiological assessment of visible capsular disruption as a key indicator of EPE [24].

Our results contrast with prior studies examining gross PPAT measurements such as total PPAT thickness or area, which have yielded inconsistent results and paradoxical inverse associations with aggressive disease in some cohorts [19]. This discordance is likely because bulk PPAT metrics fail to account for spatial relationships and tissue characteristics, specifically at the tumour capsular interface. Our focused interface approach circumvents this limitation by concentrating measurement within a narrow 3‐mm zone directly adjacent to the tumour, where biochemical and cellular cross‐talk is most likely to occur.

The clinical utility of our combined model was substantiated through DCA, which revealed positive net benefit across a wide range of threshold probabilities relevant to surgical decision‐making. Given that positive surgical margins are associated with increased biochemical recurrence and may prompt adjuvant therapy, even modest improvements in preoperative EPE prediction carry meaningful clinical consequences [25]. Furthermore, the availability of a validated nomogram facilitates integration into clinical workflows, allowing rapid risk calculation during multidisciplinary tumour board discussions or patient consultations.

A major strength of this study is the emphasis on reproducibility and clinical feasibility through simplified 2D measurements on a single axial slice. By deliberately restricting feature extraction to five interpretable parameters measurable with standard PACS tools rather than complex three‐dimensional (3D) segmentation or high‐dimensional radiomics, we avoided computational complexity and enhanced practical applicability. The excellent to good inter‐observer agreement for interface features (ICC range: 0.81–0.92) supports the reproducibility of measurements. Moreover, the streamlined workflow requiring only 3–5 min per case demonstrates practical viability for implementation in busy radiology practices without specialised software or extensive training. To facilitate reproducibility, we have included a detailed methodological figure illustrating all ROI placements and measurement techniques, enabling interested readers to replicate our approach.

Several limitations warrant acknowledgement. First, the single‐centre retrospective design limits generalisability, and external validation in independent cohorts from different institutions, geographic regions and practice settings is needed before widespread adoption. Second, although we achieved a total sample size of 240 patients with 82 EPE events, exceeding the minimum EPV criterion, larger cohorts would enable more stable coefficient estimates. Third, we did not separately analyse anterior versus posterior tumours, which may exhibit different propensities for EPE [26]. Fourth, our measurements were performed on a single axial slice rather than a volumetric analysis, which simplified the workflow but may have missed information from other levels. Fifth, our study focused on overall EPE status rather than side‐specific prediction, which would be more directly actionable for decisions about nerve sparing [27]. Sixth, the absence of external validation and prospective testing limits our ability to establish clinical impact definitively.

Despite these limitations, our findings contribute meaningful insights to the expanding literature on MRI‐based EPE prediction. Unlike many radiomics studies that extract hundreds of features requiring specialised software, our focused set of interface parameters can be measured using standard PACS tools within 3–5 min and have clear biological interpretability.

### 4.1 Future Research Directions

Future research directions include multi‐institutional external validation, integration of additional emerging biomarkers such as PSMA PET imaging features and exploration of machine learning algorithms beyond logistic regression. Given that our acquisition protocol includes high b‐value DWI (up to *b* = 2000 s/mm^2^), future studies should consider incorporating diffusion K imaging metrics, particularly K, which has demonstrated superior sensitivity to microstructural tissue variations compared with conventional ADC. Di Trani et al. [28] showed that K‐based parameters outperform diffusion tensor imaging in discriminating between benign tissue and different Gleason grades of prostate cancer, suggesting that interface K measurements may further enhance EPE prediction beyond ADC alone. Extension of our simplified 2D measurement approach to 3D volumetric analysis could potentially capture additional spatial heterogeneity. Longitudinal studies examining the relationship between preoperative interface features and post‐prostatectomy biochemical recurrence could elucidate whether interface characteristics predict not only anatomical EPE but also biological aggressiveness.

In conclusion, this study provides proof‐of‐concept evidence that simplified quantitative features of the tumour–PPAT interface measured on a single axial MRI slice using standard PACS tools enhance EPE prediction beyond standard clinical variables, with demonstrable improvements in discrimination and clinical net benefit. With multi‐institutional validation and prospective confirmation, the combined clinical–interface model could serve as a useful tool for individualised surgical planning and patient counselling in men with clinically localised prostate cancer.

## Author Contributions

Conception and design of the work: S.Z., L.H. Data collection: Z.Z., J.W., L.X., J.X., Y.X., J.L., Y.Z. Supervision: S.Z., L.H. Analysis and interpretation of the data: Z.Z., J.W., L.X., J.X., Y.X., J.L., Y.Z. Statistical analysis: S.Z., L.H. Drafting the manuscript: S.Z., L.H. Critical revision of the manuscript: all authors. Approval of the final manuscript: all authors.

## Funding

This work was supported by the Health Science and Technology Project of the Lianyungang Health Commission (202325) the Scientific Research Project of the Lianyungang Anti‐Cancer Association (MS202312).

## Ethics Statement

This study was conducted in accordance with the Declaration of Helsinki and approved by the Ethics Committee of The Second People's Hospital of Lianyungang (Approval Number: 2024K051).

## Consent

The authors have nothing to report.

## Conflicts of Interest

The authors declare no conflicts of interest.

## Data Availability

The data that support the findings of this study are available from the corresponding author upon reasonable request.
